# Body Mass Index with Tumor ^18^F-FDG Uptake Improves Risk Stratification in Patients with Breast Cancer

**DOI:** 10.1371/journal.pone.0165814

**Published:** 2016-10-31

**Authors:** Seung Hyup Hyun, Hee Kyung Ahn, Joo Hee Lee, Joon Young Choi, Byung-Tae Kim, Yeon Hee Park, Young-Hyuck Im, Jeong Eon Lee, Seok Jin Nam, Kyung-Han Lee

**Affiliations:** 1 Department of Nuclear Medicine, Samsung Medical Center, Sungkyunkwan University School of Medicine, Seoul, Republic of Korea; 2 Division of Hematology and Oncology, Department of Internal Medicine, Gachon University Gil Medical Center, Incheon, Republic of Korea; 3 Division of Hematology-Oncology, Department of Medicine, Samsung Medical Center, Sungkyunkwan University School of Medicine, Seoul, Republic of Korea; 4 Division of Breast and Endocrine Surgery, Department of Surgery, Samsung Medical Center, Sungkyunkwan University School of Medicine, Seoul, Republic of Korea; University of South Alabama Mitchell Cancer Institute, UNITED STATES

## Abstract

**Purpose:**

To investigate the combined prognostic impact of body mass index (BMI) and tumor standardized uptake value (SUV) measured on pretreatment ^18^F-fluorodeoxyglucose positron emission tomography/computed tomography (FDG PET/CT) in patients with breast cancer.

**Methods:**

We evaluated a cohort of 332 patients with newly diagnosed breast cancer (stage I-III) who underwent pretreatment FDG PET/CT followed by curative resection. Patients were categorized as overweight (BMI ≥ 23 kg/m^2^) or normal weight (BMI < 23 kg/m^2^). Primary tumor maximum SUV was measured by FDG PET/CT. Associations between BMI and tumor SUV with disease recurrence were assessed using Cox regression models.

**Results:**

Median follow-up was 39 months. There were 76 recurrences and 15 cancer-related deaths. Multivariable Cox regression analysis demonstrated that high tumor SUV (hazard ratio [HR] = 1.75; 95% CI, 1.02–3.02; *P* = 0.044) and overweight (HR = 1.84; 95% CI, 1.17–2.89; *P* = 0.008) were independent poor prognostic factors. Positive hormone receptor status was an independent predictor of favorable outcome (HR = 0.42; 95% CI, 0.26–0.68; *P* < 0.001). Overweight patients with high tumor SUV had a two-fold risk of recurrence compared to patients with normal weight or low tumor SUV after adjusting for clinical stage and tumor subtype (HR = 2.06; 95% CI, 1.30–3.27; *P* = 0.002).

**Conclusions:**

In patients with breast cancer, higher tumor SUV was associated with a more adverse outcome particularly in overweight women. BMI status combined with tumor SUV data allows better risk-stratification of breast cancer, independent of clinical stage and tumor subtype.

## Introduction

Obesity and overweight are recognized to play a prominent role in the incidence and progression of various malignancies. In breast cancer, obesity is suggested as a risk factor for cancer development [1, 2], but the association may differ according to tumor subtype and hormone dependence. A recent prospective population-based study showed an association between body mass index (BMI) and incidence of luminal type and human epidermal growth factor receptor 2 (HER2)-positive breast cancers, but not basal-like type breast cancers [3]. Other studies have shown a link between obesity and the occurrence of triple-negative [4] and hormone-negative breast cancers in younger women [5]. On the other hand, some studies failed to observe any association between BMI and breast cancer subtype [6, 7].

Obesity is not only a risk factor for breast cancer development, but also a significant prognostic factor for the disease. Hence, breast cancer patients who are overweight or obese are more likely to have poor outcome [8–13]. Suggested underlying mechanisms include increased estrogen, inflammatory cytokines, adipokines secreted by adipose tissues, and hyperinsulinemia [4, 14]. High BMI has been associated with worse outcome in hormone receptor-positive breast cancers [12, 13]. A link between high BMI and poor prognosis in triple-negative breast cancers has been shown in some studies [8, 9, 15, 16], whereas others did not observe such an association [13, 17].

The degree of tumor ^18^F-fluorodeoxyglucose (FDG) uptake on positron emission tomography with computed tomography (PET/CT) is a marker of metabolic tumor phenotype that is associated with aggressive behavior of tumor cells. In breast cancer, high tumor standardized uptake value (SUV) on FDG PET/CT is associated with poor prognostic features such as high grade, hormone receptor negativity, triple negativity, and metaplastic tumors [18–23].

Since BMI and tumor glucose metabolism are both linked to breast cancer subtypes and patient outcome, the combination of these two prognosticators may have added prognostic value to the tumor subtype according to estrogen receptor, progesterone receptor, and HER2 status. The aim of the present study was to investigate the combined prognostic impact of BMI and tumor SUV measured on pretreatment FDG PET/CT in patients with breast cancer.

## Materials and Methods

### Study Population

This study was approved by the Samsung Medical Center Institutional Review Board and the requirement for written informed consents was waived. Patient information was anonymized and de-identified prior to analysis. We retrospectively reviewed a cohort of 332 patients with newly diagnosed stage I-III breast cancer who underwent pretreatment FDG PET/CT from Aug 2006 to Dec 2012 prior to curative resection. Demographic and clinical characteristics were obtained from medical records.

Tumor subtypes were determined by means of immunohistochemical analysis for estrogen receptor, progesterone receptor, and HER2 status. HER2 staining scores of 3+ were considered positive. Tumors with a staining score of 2+ were considered HER2 positive if gene amplification was confirmed by silver or fluorescence in-situ hybridization.

BMI was defined as weight divided by the square of height, measured at the time of PET/CT. According to the criteria for Asian populations, the definitions of normal weight, overweight and obesity are BMI < 23.0, 23.0–24.9, and ≥ 25.0 kg/m^2^, respectively [24]. In this study, patients were stratified into two BMI groups, overweight/obesity (high BMI, ≥ 23.0 kg/m^2^) and normal weight (low BMI, < 23.0 kg/m^2^).

Patients were clinically follow-up every 6 to 12 months following surgery. This included history-taking, physical examination, blood carcinoembryonic antigen and cancer antigen 15–3 measurements, and radiological exams such as chest X-ray, mammography, ultrasonography and bone scintigraphy. Follow-up CT, MRI, and FDG PET/CT were performed if clinically indicated.

### PET/CT Imaging

All patients fasted for at least 6 h, and blood glucose levels were required to be less than 200 mg/dL at the time of PET/CT. Whole-body PET and unenhanced CT images were acquired using a PET/CT scanner (Discovery STE, GE Healthcare). Whole-body CT was performed using a 16-slice helical CT with 30 to 170 mAs adjusted to the patient's body weight at a 140-kVp and 3.75-mm section width. After the CT scan, at 60 min after intravenous injection of FDG (5.0 MBq/kg), an emission scan was performed from the thigh to the head for 2.5 min per frame in 3-dimensional mode. PET images were reconstructed using CT for attenuation correction with the ordered subsets expectation maximization algorithm (20 subsets, 2 iterations) with voxel size 3.9 × 3.9 × 3.3 mm. Tumor FDG avidity was measured as maximum SUV (SUVmax) normalized to patient body weight by manually placing a spherical volume-of-interest over the primary tumor.

### Statistical Analysis

Patient follow-up and survival data were obtained from medical records and the institutional tumor registry. Patients were followed-up for a median of 39 months. The primary endpoint for survival analysis was recurrence-free survival (RFS), defined as the time from pretreatment PET/CT to first occurrence of recurrent disease or distant metastasis.

Survival curves were estimated using the Kaplan-Meier method and compared by the log-rank test. Prognostic associations were assessed with univariable and multivariable Cox proportional hazards regression models. Variables for survival analyses included clinical stage, menopausal status, hormone receptor status, HER2 status, tumor SUVmax, and BMI status. The optimal cutoff for high tumor SUVmax was based on “maximally selected rank statistics” as proposed by Lausen and Schumacher [25]]. This method allows the distinction of a low and high risk group of patients by offering the selection of a cutoff point in the predictor without the problem of multiple testing. The result of the statistical analysis is shown in S1 Fig, which demonstrates maximal standardized log-rank statistics with a SUVmax cut off of 7.0. This cut off value was used to dichotomize tumor SUVmax as a variable for Cox regression and Kaplan-Meier survival analyses. All tests were two-sided and confidence intervals (CIs) were reported at the 95% level. *P* values < 0.05 were considered statistically significant.

## Results

The clinical characteristics of the patients included for analysis are summarized in Table 1. The entire study population had a mean SUVmax of 9.2 and a median of 8.15. SUVmax of the primary tumor ranged between 1.6 and 31.1. Hormone receptor-positive tumors had significantly lower SUVmax than hormone receptor-negative tumors (8.0 vs. 11.6; *P* < 0.001). Triple-negative tumors showed significantly higher SUVmax than hormone receptor-positive tumors (12.6 vs. 8.0; *P* < 0.001). Tumor SUVmax was high in 195 (58.7%) and low in 137 subjects (41.3%). The subjects were overweight in 145 cases (43.7%) and normal weight in 187 cases (56.3%).

**Table 1:** Clinical characteristics of study subjects with breast cancer (n = 332).

Characteristic	No. of patients (%)
Age at diagnosis(years), mean ± SD	46.1 ± 10.8
Menopausal status	
Premenopausal	233 (70.2%)
Postmenopausal	99 (29.8%)
Clinical stage	
I	27 (8.1%)
II	111 (33.4%)
III	194 (58.4%)
Neoadjuvant therapy	
No	132 (39.8%)
Yes	200 (60.2%)
Tumor SUVmax, mean ± SD	9.2 ± 5.6
Tumor SUVmax, median (range)	8.15 (1.6–31.1)
SUVmax > 7	195 (58.7%)
SUVmax ≤ 7	137 (41.3%)
BMI(kg/m^2^), mean ± SD	23.1 ± 3.3
Overweight (BMI ≥ 23)	145 (43.7%)
Normal weight (BMI < 23)	187 (56.3%)
Estrogen receptor status	
Negative	124 (37.3%)
Positive	208 (62.7%)
Progesterone receptor status	
Negative	144 (43.4%)
Positive	188 (56.6%)
HER2 status	
Negative	249 (75.0%)
Positive	83 (25.0%)

SUVmax, maximum standardized uptake value; BMI, body mass index; HER2, human epidermal growth factor receptor 2; SD, standard deviation.

During a median follow-up of 39 months, 76 of 332 patients (22.3%) had recurrent or metastatic disease and there were 15 cancer-related deaths (4.5%)**.** Univariable Cox proportional hazards regression analysis showed that clinical stage III, negative hormone receptor status, high tumor SUVmax, and overweight were significant prognostic factors for worse RFS (Table 2). Multivariable Cox regression analysis demonstrated that clinical stage III (hazard ratio [HR] = 2.69; 95% CI, 1.58–4.58; *P* < 0.001), high tumor SUVmax (HR = 1.75; 95% CI, 1.02–3.02; *P* = 0.044), and overweight (HR = 1.84; 95% CI, 1.17–2.89; *P* = 0.008) were independent poor prognostic factors. Positive hormone receptor status was an independent predictor of favorable outcome (HR = 0.42; 95% CI, 0.26–0.68; *P* < 0.001).

**Table 2:** Univariable and multivariable Cox regression analysis of recurrence-free survival (n = 332).

	Univariable Analysis			Multivariable Analysis		
Variable	HR	95% CI	*P*	HR	95% CI	*P*
Clinical stage III (vs. I-II)	2.69	1.58–4.57	<0.001	2.69	1.58–4.58	<0.001
Hormone receptor-positive	0.39	0.25–0.61	<0.001	0.42	0.26–0.68	<0.001
HER2-positive	0.87	0.51–1.50	0.631	0.78	0.46–1.33	0.785
Tumor SUVmax > 7	2.14	1.28–3.56	0.004	1.75	1.02–3.02	0.044
Overweight (BMI ≥ 23 kg/m^2^)	1.63	1.04–2.57	0.033	1.84	1.17–2.89	0.008
Postmenopausal status	0.86	0.52–1.43	0.575			

HR, hazard ratio; CI, confidence interval; HER2, human epidermal growth factor receptor 2; SUVmax, maximum standardized uptake value; BMI, body mass index

The 5-year recurrence rate was 27.6% in the whole population. Patients with clinical stage III at diagnosis had worse survival than those with clinical stage I-II (5-year recurrence rate, 34.1% versus 19.2%; *P* < 0.001). Patients with a high tumor SUVmax had poorer survival compared to those with a low tumor SUVmax (5-year recurrence rate, 34.3% versus 17.8%; *P* = 0.001). Overweight patients had worse survival than normal weight patients (5-year recurrence rate, 35.2% versus 22.0%; *P* = 0.021).

There was no interaction between tumor SUV and BMI. We then evaluated the combined prognostic impact of overweight with high tumor SUV after adjusting for clinical stage and tumor subtypes. Normal weight patients with low/high tumor SUV or overweight patients with low tumor SUV served as a reference group. Overweight patients with high tumor SUV had a two-fold risk of recurrence compared with the reference group (HR = 2.06; 95% CI, 1.30–3.27; *P* = 0.002).

**Fig 1:**
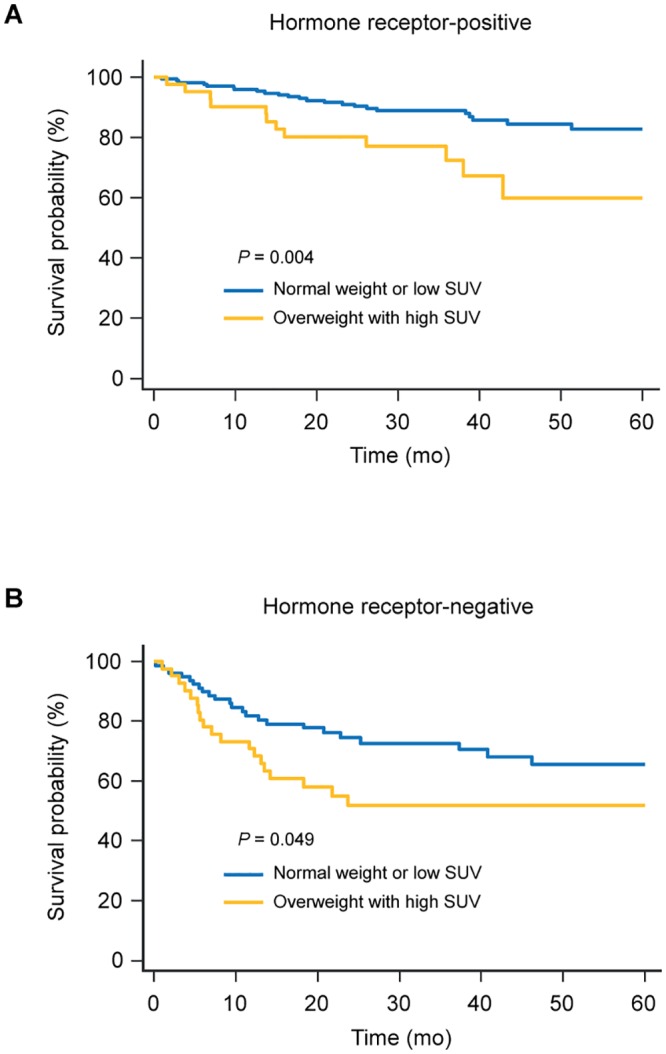
Kaplan-Meier survival curves for recurrence-free survival according to BMI with tumor SUV in patients with hormone receptor-positive (A) and-negative disease (B). BMI, body mass index; SUV, standardized uptake value.

**Fig 2:**
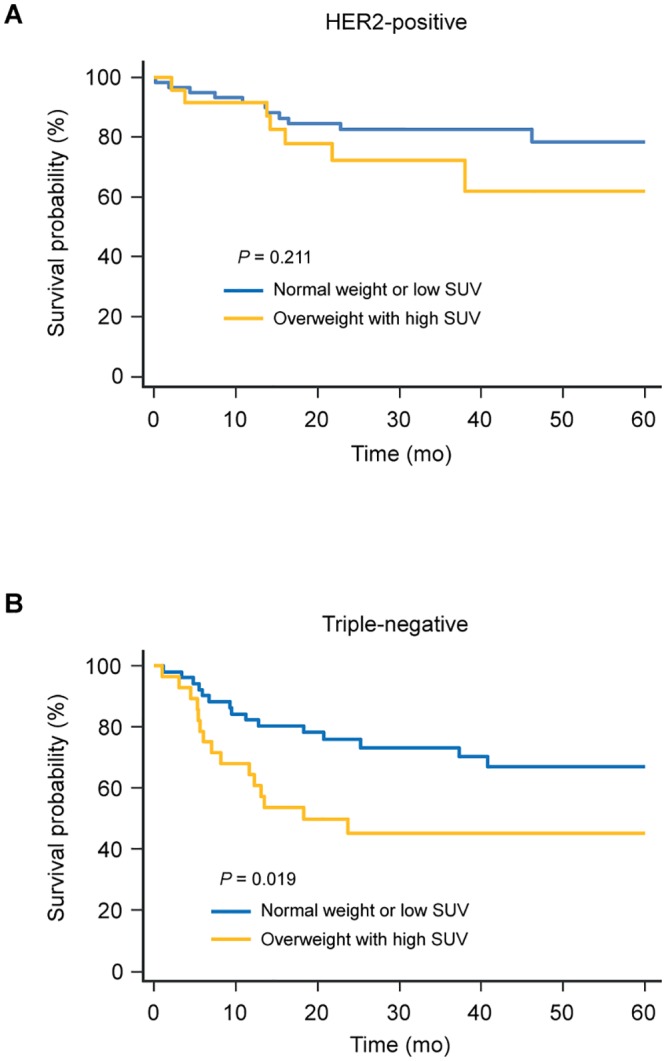
Kaplan-Meier survival curves for recurrence-free survival according to BMI with tumor SUV in patients with HER2-positive (A) and triple-negative disease (B). BMI, body mass index; SUV, standardized uptake value.

Kaplan–Meier survival analysis showed that being overweight with high tumor SUV was associated with a significantly worse survival outcome in patients with hormone receptor-positive and-negative disease, and triple-negative disease (Figs 1 and 2). In patients with HER2-positive disease, even though no statistically significant survival difference was observed, overweight women with high tumor SUV showed a worse survival outcome (Fig 2).

## Discussion

The current study demonstrated that higher tumor SUV was associated with more adverse outcome, particularly in overweight women, independent of clinical stage and tumor subtype. Patients with higher tumor SUV had a two-fold greater risk of recurrence compared to those with a lower tumor SUV. This association between high tumor FDG uptake and poor prognosis is consistent with previous studies [18, 19]. Higher SUV is linked with more aggressive features of breast cancers such as hormone receptor negativity, triple-negative subtype, and higher Ki-67 index [20–23]. Our result also shows that breast cancers with triple-negative or hormone receptor-negative subtype has higher tumor SUVmax. Breast cancer is a heterogeneous disease that consists of different intrinsic molecular subtypes with varying prognosis. In luminal B-like breast cancers, for example, low progesterone receptor expression and high Ki-67 index are suggested predictors of greater aggressiveness [26]. Our results indicate that the tumor metabolic phenotype measured on FDG PET/CT imaging may be helpful for stratifying aggressiveness among breast cancer patients.

We further evaluated the prognostic value of BMI status, and found that being overweight was a significant univariable and multivariable predictor of adverse outcome, with a 1.8-fold increase in the risk of recurrence. A previous study have demonstrated that higher BMI is independently associated with increased risk of death in hormone receptor-positive subtype of breast cancer [12]. In a clinical trial population, obesity is associated with inferior outcomes specifically in patients with hormone receptor-positive operable breast cancer [13]. Potential mechanisms for this link include increased estrogen production by adipose tissue, crosstalk between insulin or insulin-like growth factor and estrogen receptor signaling [27], obesity-associated hyper-methylation [28], and tumor growth-promoting adipokines [11, 29]. Association between obesity and poor survival outcome in breast cancer patients has been explained by predilection for advanced stage at diagnosis in obese patients. Increased lymph node metastasis and larger tumor size were found to be associated with obesity [30, 31]. However, this cannot fully explain the link since obesity was still significantly associated with poor survival after adjusting tumor stage. Under-dosing of chemotherapy in obese patients has been suggested as another explanation. This was based on the finding that first cycle dose reduction was more frequent in obese patients with breast cancer [32, 33], which was significant only in estrogen receptor-negative tumors [33]. Some studies found that obesity also predicted poor survival outcome in patients with triple-negative breast cancer, [8, 9, 15, 16], whereas other failed to observe a significant association [13, 17]. Such inconsistencies in reported relationships between breast cancer and obesity may indicate a potential role for environmental factors such as dietary habits and ethnic differences [31, 34].

A key finding in our study was that combining information of BMI status and tumor FDG uptake level allowed more powerful prediction of outcome in patients with breast cancer. Overweight women with high tumor SUV had a higher risk of recurrence following curative resection compared with patients with normal weight or low tumor SUV. This distinction suggests a potential benefit of considering patient BMI along with tumor FDG uptake level for improved risk stratification in breast cancer patients. Overweight women with high tumor SUV may be exposed to unique tumor-host environments associated with lower drug-efficacy. Therefore, such patients should be monitored closely following surgery and may be potential candidates for novel treatments.

Limitations of this study include its retrospective design, where treatment variables such as adjuvant and neo-adjuvant therapy were not controlled. In addition, all study subjects were Asians whose body composition as well as BMI criteria for being overweight and obesity are different from Western populations. Therefore, caution is warranted when applying our results to other ethnic groups. Finally, the cutoff level for high tumor SUV (SUVmax > 7) derived from the present cohort was relatively higher than the SUVmax cutoffs between 3 and 4 that were used in previous studies [18, 19, 35]. However, the median value of tumor SUV in this study cohort was 8.15 and the optimal cutoff approach was used for this study. Given the limitations of this single institution retrospective study, further external validation in a larger patient cohort will be required to assess the relevance of these findings in the management of patients with breast cancer.

## Conclusions

Higher tumor SUV was associated with a more adverse outcome in patients with breast cancer who underwent curative resection, particularly in overweight women. BMI status combined with tumor SUV allows better risk-stratification of breast cancer, independent of tumor stage and subtype. Further studies are thus needed to elucidate the underlying mechanisms for the links between BMI status, tumor glucose metabolism, and drug efficacy.

## Supporting Information

S1 Fig Optimal cutoff of SUVmax based on maximally selected rank statistics. SUVmax, maximum standardized uptake value.
(TIF)
