# Supplementary figures and images for: Body Mass Index with Tumor 18F-FDG Uptake Improves Risk Stratification in Patients with Breast Cancer
Source: PLoS One. 2016 Oct 31;11(10):e0165814. doi: 10.1371/journal.pone.0165814 (PMC5087879; doi:10.1371/journal.pone.0165814)

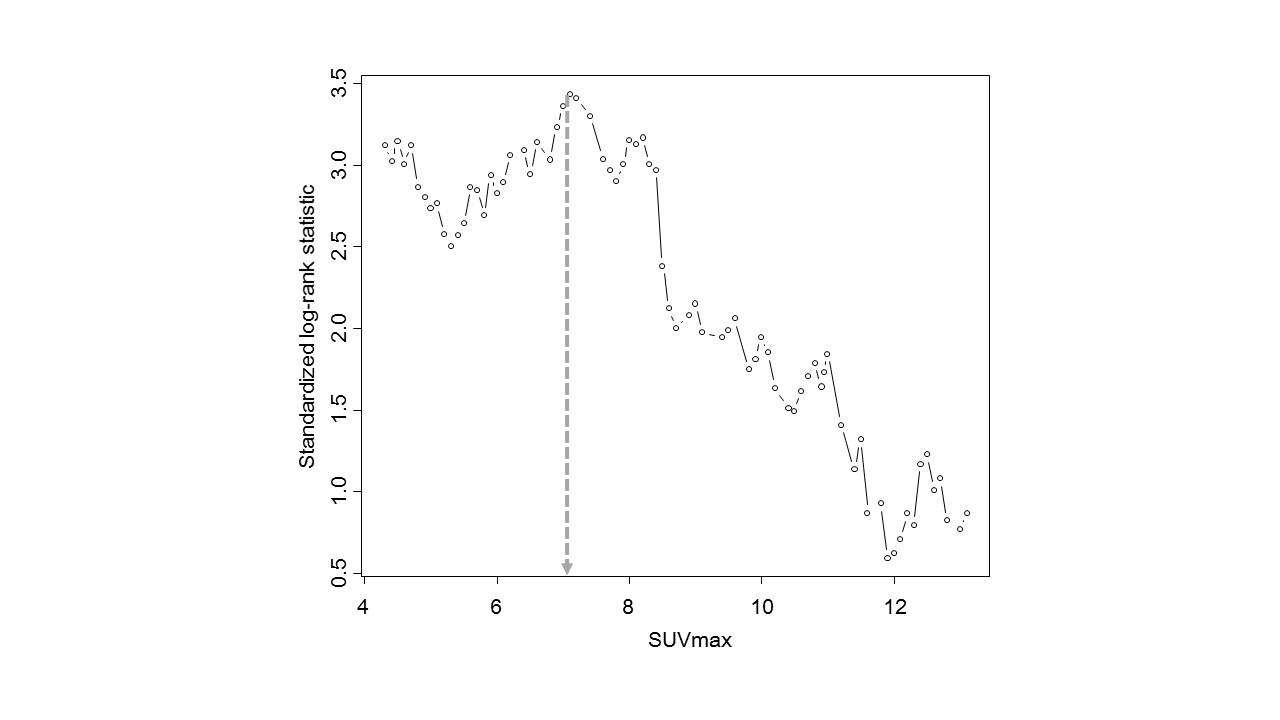

Supplement: S1 Fig — SUVmax, maximum standardized uptake value. (TIF) [file pone.0165814.s001.tif]
